# The COVID-19 pandemic: an increased risk of rheumatoid arthritis

**DOI:** 10.2217/fvl-2020-0393

**Published:** 2021-06-22

**Authors:** Archana Tripathy, Nitish Swain, Bhawna Gupta

**Affiliations:** ^1^Disease Biology Laboratory, School of Biotechnology, Kalinga Institute of Industrial Technology (KIIT) deemed to be University, Bhubaneswar, Odisha, 751024, India

**Keywords:** angiotensin-converting enzyme 2, angiotensin II, rheumatoid arthritis, SARS-CoV-2, Th1/17 response

## Abstract

COVID-19 is a respiratory infection similar to viral pneumonia and is caused by SARS-CoV-2. Chloroquine and hydroxychloroquine make up the major part of the treatment regimen for the management of COVID-19 infections, which are also commonly used in treatment of patients with malaria as well as autoimmune diseases like rheumatoid arthritis (RA). In this review, we analyzed the scientific evidences pertaining to any possible association of SARS-CoV-2 infection with RA. We thus believe that people predisposed to RA carry a higher infection risk than the general population both due to the iatrogenic effects of the RA related drug therapy. Thus COVID-19 pandemic may bring a higher risk of health emergency in complex diseases such as RA.

The year 2020 unfortunately has seen a dark beginning with the spread of a novel coronavirus pandemic COVID-19. Alhough the disease initiated from the Wuhan province of China, it has now spread to more than 200 countries worldwide. As of 21 June 2021 COVID-19 has affected more than 177 million people worldwide with above 3.8 million deaths. The countries have locked down international borders, asked residents to stay home and work from home with only essential services running. Amidst the uncertainty people suffer from economical downfall, anxiety and stress while COVID-19 epidemiology is rapidly evolving with an increasing number of cases on a daily basis.

Although a number of coronaviruses of the family Coronaviridae have been known to infect humans [1] yet the infections have been mild and resulted in common cold and flu-like symptoms. New and pathogenic strains of these viruses (MERS-CoV, SARS-CoV) have emerged with high fatality rates resulting in acute respiratory distress syndrome, reduced lung function, arrhythmia and eventually death. COVID-19 is known to be caused by a mutated strain of SARS-CoV that is reported to be enveloped with a positive-sense ssRNA genome and hence named SARS-CoV-2. The human angiotensin-converting enzyme 2 (ACE2) is identified as the host cell-surface receptor for the envelope spike glycoprotein of SARS-CoV-2, facilitating its entry and infection in the host cell [2]. The ACE2 is a cell membrane receptor expressed on a number of different types of cells including the cells of the GI tract, blood vessels, lung AT2 alveolar epithelial cells and others [3]. The interaction of ACE2 with SARS-CoV-2 spike protein results in ACE2 downregulation, an increased production of angiotensin II, a subsequent activation of type 1a angiotensin II receptor (AT1RA) that increases pulmonary vascular permeability thereby increasing lung damage [4,5]. COVID-19 thus results in a chronic condition and hence a pandemic situation has arisen. The COVID-19 is also detrimental to the debilitating population with autoimmune rheumatic diseases that are either at the higher risks of infection leading to disease severity or may suffer due to the induced effects of the immune-suppressive agents like the disease-modifying antirheumatic drugs.

Rheumatoid arthritis (RA) is one of the most prevalent and debilitating autoimmune diseases characterized by the inflammation of the synovium [6,7]. The patients suffer from acute pain and swelling of joints such that they are unable to move, walk, hold objects and thus become dependent on others for their daily activities. Although etiology of RA is unknown yet it is linked to several genetic and environmental factors [8,9]. This disease condition is marked by presence of a dysfunctional immune system that recognizes self antigens as foreign. The preclinical phase is said to be characterized by the generation of auto antibodies and the clinical phase is attained when the body reacts to these auto-antibodies leading to inflammation [8]. The inflammatory condition is driven by the cytokines that are upregulated during the disease condition. Th1cells producing IFN-γ and Th17 cells producing IL-17 are the key regulators for disease progression [10,11]. The release of granzyme and perforin by the activated CD8^+^ T cells is also known to aggravate the disease condition [12]. The similarities in the cytokine profile, lymphocyte population characteristics and inflammatory mediators interestingly present an intricate relationship between COVID-19 and RA. We thus have tried to find potential reasons of co-existence of COVID-19 and RA as well as the repercussions of COVID-19 pandemic in the increase in population of RA patients (Figure 1) worldwide.

**Figure 1.**
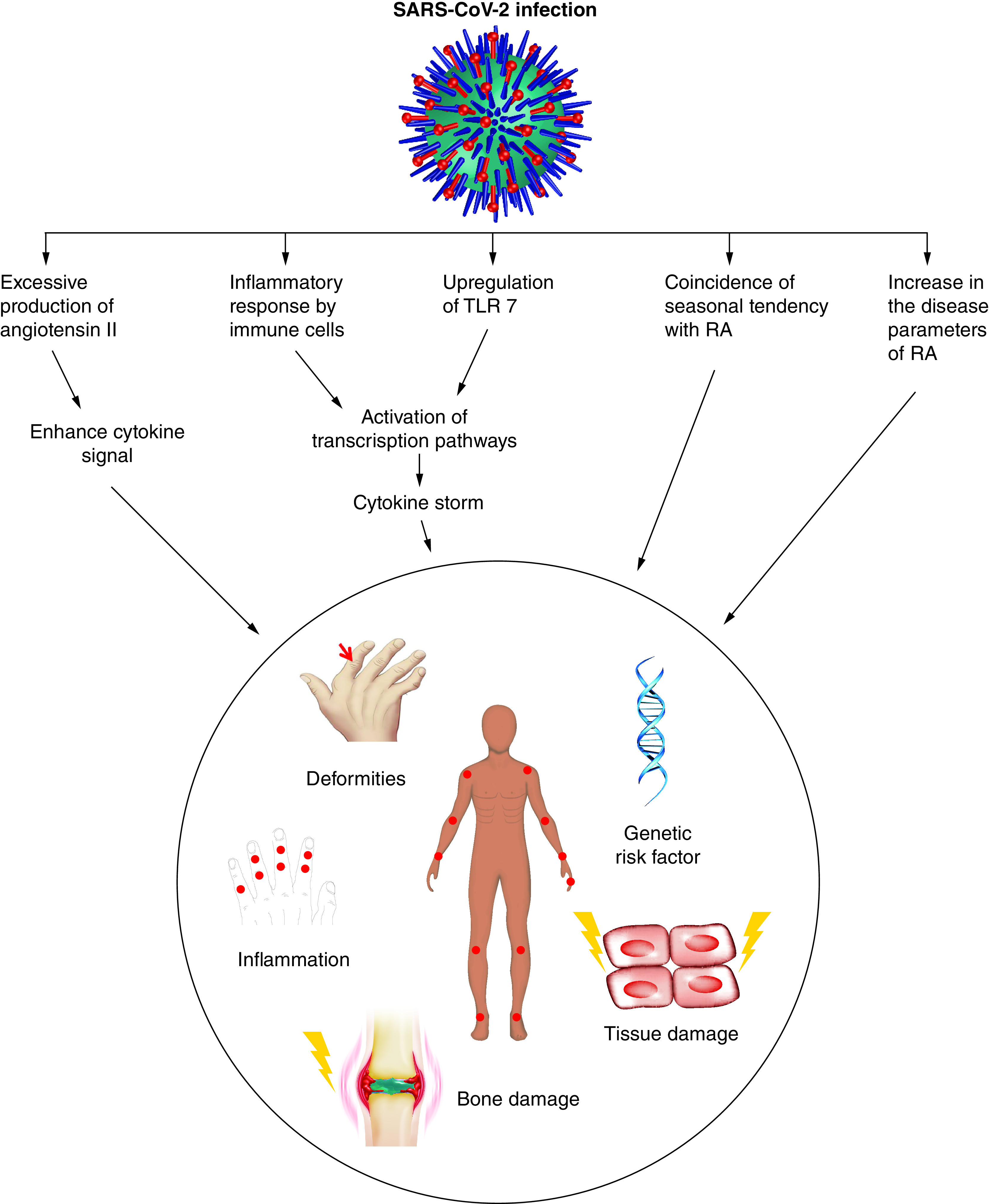
SARS-CoV-2 infection can potentiate initiation, progression and perpetuation of rheumatoid arthritis using parallel mechanisms. RA: Rheumatoid arthritis.

## Methodology

A nonsystematic review for published literature was carried out in PubMed and Google Scholar databases. Articles published in 2020 having terms “SARS-CoV-2” OR “COVID-19” OR “Rheumatoid Arthritis” in the title were considered.

In this study the inclusion of articles was done on the model proposed by the User’s Guide to Medical Literature (JAMA Network).

## Excessive production of angiotensin II by SARS-CoV-2 infection

**Figure 2.**
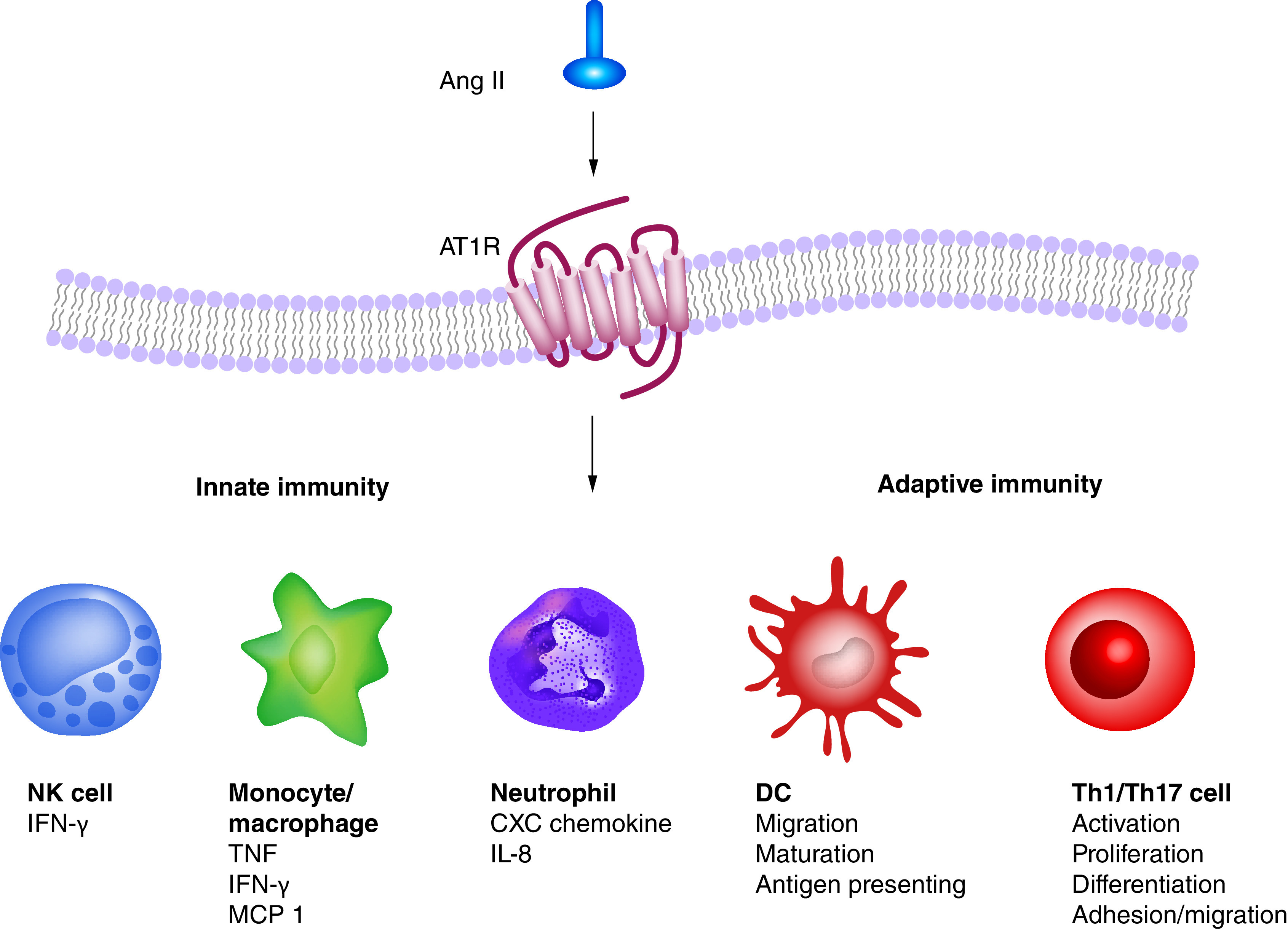
The inflammatory roles of angiotensin II on innate and adaptive immune response. Ang II via AT1R signaling in immune cells contributes to the secretion of pro-inflammatory mediators and enhances inflammatory responses by activating innate and adaptive immune cells. Ang II: Angiotensin II; DC: Dendritic cell; NK: Natural killer.

SARS-CoV-2, a positive-sense ssRNA virus, has the genome size of 26–32 kb, a crown-like appearance under an electron microscope with a confirmed presence of spike glycoprotein on the envelope [1]. Two-thirds of the genome open reading frame toward 5’ terminal encodes replicase transcriptase complex and rest of the genome encodes the structural proteins including spike (S), envelope (E), nucleocapsid (N) and membrane (M) proteins [13]. The entry of corona virus into the host cells is a result of attachment of spike (S) protein to host cellular receptors called ACE2 [4,14,15] resulting in an increased expression of serum angiotensin II [5]. Angiotensin II is recognized as a powerful pro-inflammatory mediator that acts through stimulation of angiotensin II receptor (AT1R), present on the cell surface of lymphocytes, macrophages and other immune cells [16,17]. The presence of angiotensin II receptor and its elevated expression in the synovium of RA patients leads to synovial hyperplasia [18,19]. Kawakami *et al.* showed that the cytokines like IL-1 regulates the expression of both the angiotensin II receptors, AT1R and AT2R, in articular chondrocytes in RA patients [20]. AT1R antagonist, losartan reduces the level of C-reactive protein (CRP) and erythrocyte sedimentation rate of RA patients [21]. An *in vitro* study showed that the losartan treatment suppressed TNF-α production in synovium of inflamed joints of RA patients [19]. Excessive angiotensin II concentration initiates inflammatory response by stimulating the production of VEGF and prostaglandins as well as increasing vascular permeability [22]. Angiotensin II as well activates monocytes and upregulates the expression of TNF-α, IL-6 and IL-8, which are the potent activators of neutrophils [23]. In addition, it also increases CRP transcripts and protein in macrophages via NF-κB activation and angiotensin receptor mediated reactive oxygen species (ROS) production [24]. angiotensin II can directly enhance the production of ROS in leucocytes and stimulate the activation of lymphocytes [25]. Therefore, angiotensin II signaling through AT1R subsequently produces pro-inflammatory cytokines and chemokines by peripheral blood cells which contributes to the migration of immune cells to the site of injury and multiplies the inflammatory response (Figure 2) [26]. Above reports may potentially explain that the genetically predisposed individuals for RA if infected with SARS-CoV-2 may suffer from the disease initiation, while the already existing RA patients may see a surge in the disease severity. Also the excessive presence of angiotensin II due to the infection of SARS-CoV-2 will stimulate AT1R and AT2R, which may aggravate symptoms and severity of RA.

## Cellular immune response by SARS-CoV-2 infection

Innate immune cells are important target cells of angiotensin II and express both AT1R and AT2R, which have substantial roles in production of metalloproteinase and promoting vascular inflammation. Xu *et al.* recently performed peripheral blood cell profiling of COVID-19 patients by flow cytometry [27]. They found the number of CD4^+^ as well as CD8^+^ T cells declined significantly in SARS-CoV-2 infected patients, but the cells were highly activated, which was confirmed by the presence of high proportions of HLA-DR (CD4: 3.47%) and CD38 (CD8: 39.4%) double positive populations. Increased concentration of pro-inflammatory CCR6^+^ Th17 subpopulation of CD4^+^ T cells was also identified [27]. Additionally, CD8^+^ T cells were found with higher concentration of cytotoxic granules wherein 64.2% cells were granulysin positive, 31.6% cells were perforin positive while 30.5% cells were both perforin and granulysin positive [27]. This data imply that over activation of T cells, increased percentage of Th17 cells and high cytotoxic activity of CD8^+^ T cells all together leads to severe immune injury and may increase the chance of incidence of T lymphocyte mediated diseases like RA.

Angiotensin II acts as a co-stimulatory signal for activation, differentiation and proliferation of T cells via AT1R [28,29]. Angiotensin II induces Th1 differentiation by increasing the expression of IFN-γ in addition with reduction of IL-4 [29]. It also stimulates the production of two prominent inflammatory cytokines, IFN-γ and TNF-α, in T cells (Figure 2) [30]. Commercially available AT1R blockers like olmesartan, telmisartan and candesartan have also been shown to inhibit antigen specific Th1 and Th2 immune responses [31]. Wu *et al.* demonstrated that angiotensin II also induces Th17 responses [32]. Th1 and Th17 differentiation by angiotensin II thus suggests progression of RA upon SARS-CoV-2 infection.

## Involvement of Toll-like receptor during coronavirus infection

TLR7, an endosomal receptor for ssRNA, is present on the endosomal membrane [33,34]. It can differentiate self RNA from nonself viral ssRNAs due to the presence of higher number of modified nucleotide bases (m5C, m5U, m6A and s2U) in cellular RNA [35]. TLR7 is predominantly expressed by RA synovial macrophages and by RA synovial fibroblasts cells [36]. Expression of TLR7 is also upregulated in RA monocytes and shows a strong positive correlation with TNF-α levels as well as DAS28 [36]. It has been reported that the SARS-CoV infection induces upregulation of TLR7 in monocytes (THP1 cells) however the downstream signaling mechanism is not clear till date [37]. TLR7 signaling promotes the transcription of cytokines involved in Th17 cell differentiation as well as inhibits the TGF-β signaling [38,39]. TGF-β is widely recognized for its role in reducing inflammation and maintaining pulmonary homeostasis by inhibiting both Th2 and Th1 responses [40,41]. It thus becomes imperative to understand if the increased TLR7 in RA patients can efficiently recognize ssRNA of SARS-CoV-2 and initiates the TLR7 mediated inflammatory response further potentiating disease severity.

## Cytokine storm in COVID-19

**Figure 3.**
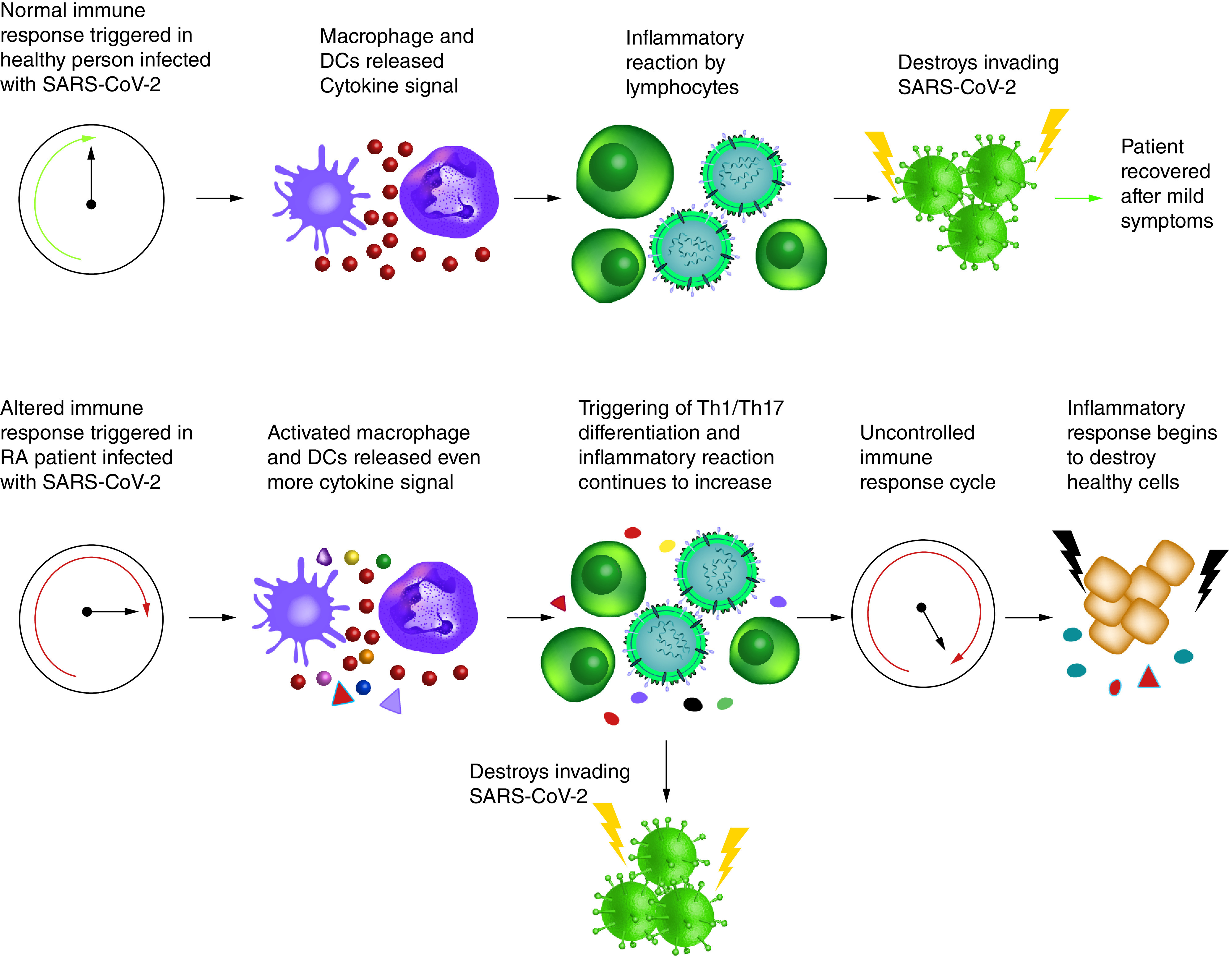
Schematic diagram represents the comparison of inflammatory responses by a healthy individual with those by a rheumatoid arthritic patient when infected with SARS-CoV-2. Cytokine storm causes uncontrolled immune response cycle in rheumatoid arthritic patients and begins to destroy healthy/self-cells. But in case of healthy individuals infected with SARS-CoV-2, they may recover with mild symptoms after a series of inflammatory reactions by innate and adaptive immune cells. RA: Rheumatoid arthritis.

COVID-19 is considered as cytokine storm syndrome because of robust increase in several cytokines and chemokines in addition to the cytolytic enzymes (Figure 3). Patients with novel COVID-19 show elevated serum levels of IFN-γ, TNF-α, IFN-γ-induced protein 10 (IP 10), monocyte chemo attractant protein-1 (MCP-1), macrophage inflammatory proteins (MIP) which belongs to the family of chemotactic cytokines known as chemokines, granulocyte-colony stimulating factor, granulocyte-macrophage colony-stimulating factor (GM-CSF), IL-1β, IL-2, IL-7, IL-8, IL-9, IL-17 and others [42,43]. Cytolytic enzymes like perforin and granulysin are also increasingly secreted by CD8^+^ T cells of COVID-19 patients [27].

Among these, several cytokines are involved in inducing Th1 and Th17 type responses (Figure 3). TNF-α and IL-1β both induce Th17 lineage differentiation of naive CD4^+^ T cells and promote vascular permeability and leakage [44]. TNF-α is concomitantly highly expressed by both Th1 and Th17 cells [45]. Th17 cells secrete IL-17 and GM-CSF [46,47]. IFN-γ and GM-CSF secretions are also associated with Th1 cells in humans [48,49]. IL-21 is required for Th17 cell maintenance in a STAT-3 dependent manner [50]. IL-17 has broad pro-inflammatory effects on induction of cytokines like TNF-α, GM-CSF, IL-1β and IL-6 (induce systematic inflammatory response); chemokines like IP10, MIP2A, IL-8, MIP3A (attract and recruit immune infiltrates) and matrix metalloproteinases (MMP) (participate in tissue damage and remodeling) [32].

RA is known to be a Th1/Th17 disorder wherein naive CD4^+^ T cells increasingly differentiate into Th1 and or the Th17 lineage. Several pro-inflammatory mediators known to be responsible for an increased differentiation of naive CD4^+^ T cells to Th1/Th17 phenotype is found to be increased in COVID-19 patients. Taken together, we thus propose that cytokine storm developed by Th1 and Th17 type responses during SARS-CoV-2 infection may increase the incidence of RA and trigger a violent attack by the immune system thereby making it difficult for the patient to recover from the SARS-CoV-2 infection.

## Pathways associated with SARS-CoV-2 infection

Several cytokines including IFN-γ, GM-CSF, IL-6 and IL-15 involved in RA pathogenesis activate janus kinase/signal transducer and activator of transcription (JAK/STAT) pathway [51]. STAT-3 is constitutively activated in RA patients [51]. Due to excessive presence of IFN-γ and GM-CSF in the serum of a COVID-19 patient [27], these can activate JAK/STAT pathway and lead to the upregulation of several interferon stimulated genes [52] which can elevate the amount of cytokines having the ability to attack self cells and induce inflammation in RA. JAK/STAT mediated activation of immune cells by SARS-CoV-2 infection may thus contribute in severity and progression of RA. It is already known that angiotensin II level is high in COVID-19 patients and its signaling through AT1R leads to activation of NF-κB with subsequent synthesis of pro-inflammatory cytokines, chemokines, cytolytic enzymes and cell adhesion molecules [53,54]. They contribute to the migration of immune cells to the site of infection and amplify the inflammatory responses. MAPK pathways comprises of the p38 mitogen activated protein kinases (MAPK), extracellular signal regulated kinase (ERK) and janus kinase (JNK) pathways [55]. During SARS-CoV and MERS-CoV infections, the signals are transmitted by a series of protein kinase cascades of MAPK pathways, with phosphorylation at Tyr and Thr residues [56]. During coronavirus infection, viral protein triggers the activation of MAPK pathways, which in turn regulate the production of variety of cytokines [57]. All these intermediates of transcription pathway associated with SARS-CoV infection collectively produce inflammatory cytokines, which can increase the incidence of infection in RA and may progressively increase the disease severity in RA patients.

## Other related consequences of SARS-CoV-2 infection in RA

### Changes in clinical parameters

RA is characterized by perturbations in the levels of different clinical parameters like erythrocyte sedimentation rate (ESR), CRP, anti-CCP and rheumatoid factor. Recent reports of COVID-19 suggest significant upregulation in the levels of ESR and CRP post infection [58]. Studies with SARS-CoV have revealed an increase in CRP levels in the affected population, whereas in MERS-CoV rise in both CRP and ESR levels have been reported [59–61]. Respiratory viral infection studies have also revealed higher expression of CRP and ESR [62,63]. Thus, excessive secretion of angiotensin II post infection by SARS-CoV-2 increases two specific disease parameters of RA namely ESR and CRP [21].

### Genetic factors

RA is classified as HLA II (MHC II) associated disease and *HLA-DRB1* alleles are the most relevant genetic factors for disease susceptibility [64]. The antigen presentation of MERS-CoV infection mainly depends on *MHC II* molecules [65], whereas SARS-CoV depends on both *MHC I* and *MHC II* molecules [66]. As the mechanism of antigen presentation of SARS-CoV-2 is still unclear, we can predict a genetic predisposition toward COVID-19 in RA.

### Clinical studies

The clinical studies that have taken place in the past year on COVID-19 and RA are mostly case studies. The case studies have mostly focused on the levels of markers of RA in people who have encountered COVID-19. Increase in ESR, Anti-CCP, IL-6 and CRP has been reported in patients who had been affected by COVID-19 with history of RA [67–69]. These signature clinical parameters have also been reported to increase post COVID-19 infection and patients have shown symptoms of early onset of RA. Presence of anti-PAD2, anti-PAD4 along with Sjogren syndrome related antibodies have been reported too [69]. Patients with remising RA were seen to have a flare in disease activity post infection with COVID-19 [70].

### Effects of biologic disease-modifying anti-rheumatic drugs

Biologic treatments have been in use for the treatment of RA due to their less side effects as compared with conventional disease-modifying anti-rheumatic drugs (DMARDs). The mostly used biologic disease modifying anti-rheumatic drugs (bDMARDs) are tocilizumab, eternacept, anakinra and barcitinib. In case of RA these bDMARDs act as IL-6, TNF-α, JAK and IL-1 blockers, respectively and bring down the production of cytokines though rendering the patient immunosuppressed [71]. In case of COVID-19 the initiation of cytokine release syndrome takes place in severe COVID-19 infection. These bDMARDs have been introduced as a supplementary treatment regimen along with the use of antivirals. When administered these bDMARDs block their specific targets and help in relieving the cytokine load on the infected patient thereby partially eliminating the risk of cytokine release syndrome [72,73]. The main drawback of this therapy is the state of immunosuppression that occurs in the patient which may lead to several opportunistic infections. Research and case studies have proposed the supplemented use of these biologic DMARDs in combination with antivirals and with proper timely assessment of the patient to look for the opportunistic infections [72,74].

### Recurrence of COVID-19 & similarities with recurrence of RA

The sequel or recurrence of COVID-19 has mostly been seen in older patients and can be attributed to numerous factors. The patient post recovery has a very poor physical health, poor resistance and impaired physical functions [75]. The poor immune function after the duration of treatment also increases the chances of re-infection. The treatment regimen includes use of glucocorticoids which inhibit the body immune function and increase the risk of secondary infection [76]. Co-morbidities associated with the patient are also a major contributing factor toward the recurrence of COVID-19. The recurrence of COVID-19 is similar to the relapse of RA in the aspects of age, physical health status and physical function. RA relapse mostly occurs in patients who have had the disease for long periods of time and with old age the body being immune suppressed acquires other infections. The associated co-morbidities like Type 2 diabetes mellitus (T2DM), interstitial lung disease (ILD) and osteoporosis also trigger the relapse of RA [77].

### Diagnostic findings

As of now the gold standard for diagnosis of COVID-19 is the real time polymerase chain reaction (RT-PCR) test. The lack of diagnostic findings in both diseases are due to their complicated natures. COVID-19 being a viral infection has high possibilities of mutations and in case of RA the etiology is still unknown [78]. The specific reason for the onset of RA is yet to be elucidated. Current test for RA diagnosis is based on the levels of various inflammatory markers like complete blood count, CRP and serum glutamic pyruvic transaminase and these markers are also monitored in COVID-19 to check for the onset of cytokine release syndrome [79]. To increase the diagnostic findings in case of both the diseases we have to focus our research on the etiology and with this find out novel markers for better treatment of both the diseases.

### Preventive medication

The use of chloroquine derivatives such as hydroxychloroquine was started due to its antiviral and anti-inflammatory properties but the major pushing force was its availability and the inexpensive nature of the drug. Hydroxychloroquine modulates the infection by acting on the viral entry mechanism. It has been shown to make the ACE2 receptor malfunction and hence treat the patients [80].

But the use of hydroxychloroquine showed lack of preventive role because this drug failed to act on the viral replication. It was seen that in comparison to other antivirals, hydroxychloroquine did not prove beneficial [81]. As the treatment required high doses of hydroxychloroquine certain side effects including gastrointestinal symptoms, pruritus and dermatological changes which have further discouraged its use for treatment of COVID-19 [82].

## Conclusion

COVID-19 has presented itself as the greatest global crisis of this millennium and has affected almost all the countries in the world. Research on the pathophysiology of COVID-19 has brought into light different aspects of this novel disease. The increased levels of angiotensin II which is one of the key characteristic of the disease has been shown to up regulate the levels of pro-inflammatory cytokines like TNF-α, IL-6 and IL-8 which are significantly increased in case of RA [83,84]. Angiotensin has also been correlated with the production of IFN-γ which is a key cytokine in case of RA. The increase in the level of CRP can be seen as a link to the potential occurrence of RA in a COVID-19 patient [24,25,85]. High ROS production and activation of NF-κB are some of the other factors that may govern the incidence of RA [25]. The highly activated lymphocytic population and Th1/Th17 responses post infection with SARS-CoV-2 can be seen as a key factor for the occurrence of RA in COVID-19 patients [86]. The activation of JAK/STAT, NF-κB and MAPK pathways during the SARS-CoV infection resulting in the synthesis of several inflammatory mediators might just trigger the genetic factors associated with RA or might lead to an aggravated immune response [27]. The suppressed immune system in case of RA, due to the use of conventional and biologic DMARDs may present complications if infected with SARS-CoV-2. This may prolong the onset of cytokine release syndrome but may open up the victim to other opportunistic infections that might increase morbidity. There have been studies where the relation between other respiratory viral infections and RA has been shown. Thus, we propose that RA patients might be predisposed to disease severity post SARS-CoV-2 infection suggesting that severe RA and COVID-19 might coexist and the genetically predisposed individuals to RA may have a high risk to develop RA if they get infected with SARS-CoV-2.

## Future perspective

For a better understanding of SARS-CoV-2 we need a series of strategic research especially with respect to RA. RA being a complicated auto-immune disorder will only add to the cytokine burden in a patient with SARS-CoV-2 infection. The information obtained while analyzing the case studies with RA patients suffering from SARS-CoV-2 must be made available to clinicians worldwide for the better management of the disorder.

**Executive summary**

- In this review we have tried to find potential reasons of co-existence of COVID-19 and rheumatoid arthritis (RA) as well as the repercussions of COVID-19 pandemic in the population of RA patients worldwide.

**Excessive production of angiotensin II by SARS-CoV-2 infection**

- The entry of SARS-CoV-2 is a result of attachment to host cellular receptor angiotensin-converting enzyme 2. The presence of angiotensin II in RA initiates inflammatory response by activation of monocytes and upregulates the expression of TNF-α, IL-6 and IL-8. Increase in C-reactive protein (CRP) and reactive oxygen species also help in activation of lymphocytes. Therefore, Angiotensin II signaling through AT1R subsequently leads to the production of chemokines and pro-inflammatory cytokines and contributes to the activation and migration of immune cells to the target site and elicits the immune response.

**Cellular immune response by SARS-CoV-2 infection**

- The increase in the cytotoxic activity of the CD8 + T cells has been seen in SARS-CoV-2. The increase in Th17 fate of immune cells might increase the chances of a Th17-mediated disease like RA. Angiotensin II has also been found to act as a co-stimulatory signal for activation, differentiation and proliferation of T cells via AT1R, leading to production of IFN-γ and TNF-α. This Th1 and Th17 fate of the T cells suggest onset or progression of RA upon SARS-CoV-2 infection.

**Involvement of Toll-like receptor during coronavirus infection**

- TLR7 is an endosomal receptor present in immune cells and recognizes ssRNA. The recognition of SARS-CoV-2 genetic material by TLR7 might lead to a Th17-driven immune response, RA being a Th17-driven disorder might become more aggravated upon infection with SARS-CoV-2. The downstream events occurring must be studied carefully to know he interaction that occurs between SARS-CoV-2 viral genome and if that can lead to an aggravated form of RA.

**Cytokine storm in COVID-19**

- Cytokine storm is seen in patients with very severe cases of COVID19, robust increase in levels of IFN-γ, TNF-α, macrophage inflammatory proteins, granulocyte-macrophage colony-stimulating factor, IL-1β, IL-2, IL-7, IL-8, IL-9, IL-17, perforin and granulysin has been reported. These inflammatory molecules play a major role in the disease progression in a patient suffering from RA. The onset of such cytokine storm might lead to the onset or progression of RA.

**Pathways associated with SARS-CoV-2 infection**

- Cytokines like IFN-γ and granulocyte-macrophage colony-stimulating factor which are highly activated in RA are potent activators of JAK/STAT pathway. COVID-19 also presents expression of these cytokines in large quantities, this may lead to the activation of the JAK/STAT pathway and induce inflammation. The AT1R signaling leads to activation of NF-κB which results in the synthesis of numerous inflammatory molecules.

**Other related consequences of SARS-CoV-2 infection in RA**

- RA is characterized by perturbations in the levels of different clinical parameters like ESR, CRP, Anti-CCP and rheumatoid factor. Recent reports of COVID-19 suggest significant up regulation in the levels of ESR and CRP postinfection. Genetic factors like HLA II (MHC II) associated disease and HLA-DRB1 alleles are the most relevant genetic factors for disease susceptibility. The antigen presentation of MERS-CoV infection mainly depends on MHC II molecules, whereas SARS-CoV depends on both MHC I and MHC II molecules. As the mechanism of antigen presentation of SARS-CoV-2 is still unclear, we can predict a genetic predisposition toward COVID-19 in RA. Biologic DMARDs have been introduced as a supplementary treatment regimen along with the use of antivirals. When administered these bDMARDs block their specific targets and help in relieving the cytokine load on the infected patient thereby partially eliminating the risk of cytokine release syndrome.

**Future directions**

- Research needs to focus on large cohorts rather than individual case studies. This would enable the researchers to find out the key aspects of COVID-19 and RA relationship.
